# Blood Pressure Classification Using the Method of the Modular Neural Networks

**DOI:** 10.1155/2019/7320365

**Published:** 2019-01-23

**Authors:** Martha Pulido, Patricia Melin, German Prado-Arechiga

**Affiliations:** ^1^Tijuana Institute of Technology, Calzada Tecnológico, Tijuana 22379, Mexico; ^2^Cardiodiagnostico Excel Medial Center, Tijuana 22010, Mexico

## Abstract

In this paper, we present a new model based on modular neural networks (MNN) to classify a patient's blood pressure level (systolic and diastolic pressure and pulse). Tests are performed with the Levenberg-Marquardt (trainlm) and scaled conjugate gradient backpropagation (traincsg) training methods. The modular neural network architecture is formed by three modules. In the first module we consider the diastolic pressure data; in the second module we use details of the systolic pressure; in the third module, pulse data is used and the response integration is performed with the average method. The goal is to design the best MNN architecture for achieving an accurate classification. The results of the model show that MNN presents an excellent classification for blood pressure. The contribution of this work is related to helping the cardiologist in providing a good diagnosis and patient treatment and allows the analysis of the behavior of blood pressure in relation to the corresponding diagnosis, in order to prevent heart disease.

## 1. Introduction

The learning ability of neural networks and their pattern classification characteristics are the reasons why these models can be of great importance for medical applications. Nowadays there are many approaches in intelligent computing, such as evolutionary computing, fuzzy systems [1–7] and neural networks [8–18], which are used in the areas of medicine [17–22].

Hypertension that threatens to be present in most of the people of the world is a dangerous disease and leads to fatal consequences such as death and is a risk factor for people who suffer from it: obesity, diabetes mellitus, etc.

Hypertension is a global problem as it affects more than a billion people and causes more than ten million (avoidable) deaths each year. The only way to know if a person suffers from this disease is to constantly check the blood pressure and effectively diagnose and prevent this disease [23, 24].

Currently, there are several computer techniques that have been applied in medicine, such as neural networks and fuzzy systems to diagnose hypertension; by using these methods we can provide information about the factors and risks the patient may have.

The main contribution of this work is the proposed Arterial Hypertension Classification and Diagnosis model based on modular neural networks for disease prevention. In this way, the cardiologist with the help of the model may prescribe the necessary treatment to the patient since hypertension is a disease that can evolve without showing any symptoms; this is the reason it is also known as “the silent killer”.

In this work, an MNN model is used to classify the patient's hypertension level, tests are also performed with the Levenberg-Marquardt (trainlm) and scaled conjugate gradient backpropagation (traincsg) training methods, and this neural network consists of three modules. In the first module we consider the diastolic pressure data; in the second module we use details of the systolic pressure; in the third module, pulse data is used. Therefore, we obtain the patient's blood pressure through ambulatory blood pressure monitoring (ABPM); so far we have 300 records.

### 1.1. Overview of Related Works

Artificial neural networks with the back propagation learning algorithm to obtain the hypertension diagnosis were presented by Sumathi B. et al. [25]. The method was designed with eight risk factors: smoking, stress, family history, high cholesterol, etc., where the result of neural network classification shows if the patient suffers from arterial hypertension.

Huang S. et al. [26] presented a study to investigate the factors of Hypertension (HTN) and was implemented as a prediction model for 35-year-old people in a rural area of China, with a modular neural network, considering risk factors, such as socioeconomic status and education level.

Vilkov V.G. et al. [13, 27] presented a comparative study with models of daily blood pressure monitoring was performed in 34 apparently healthy subjects and 72 patients with arterial hypertension (AH). They compared the efficiency of diagnosis of latent AH using models based on artificial neural networks of different architectures.

Barman M. et al. [28] presented an intelligent system based on a fuzzy rule system, to diagnose heart diseases and the number of heart attacks; such fuzzy system has seven inputs and uses the Cleveland database.

Patil P. et al. [29] designed a sensor which measures the pulse and temperature of the patient and is controlled by a fuzzy system which shows the patient's pulse via remote and sends a warning to relatives, doctors, or ambulances, in case it presents an emergency for patients.

Morsi I. et al. [30] presented a model to diagnose blood pressure. A group of 105 patients is used to design this model and another group with the same number of patients is used to test and thus check the efficiency of fuzzy systems in the field of medicine.

Hussein S. [31] analyzed the risk factors of hypertension and a model was designed for the prediction of rural residents over 35 years of age, considering several factors such as education level, sedentary work, and history of hypertension in the family.

Touyz M. R. et al. [32] presented an ANFIS system; methodology is designed to diagnose and compare an existing fuzzy expert system, regarding performance metrics accuracy and sensitivity.

### 1.2. Artificial Neural Networks

Neural networks are integrated by many interrelated components. A neural network can have a structure of multiple inputs and outputs; these systems operate similarly to the human brain. A neural network learns from input values, and this helps us learn about the human being [33–35].

### 1.3. Hypertension

In Mexico, a large number of professional studies have been made with the idea of determining the prevalence of hypertension, defined as the frequency of the disease at a given time in a particular place. The most important studies use different methodological criteria, which make them difficult to compare. In these studies, different blood pressure levels are used to define this disease. They even propose an adequate standardization to measure it, which usually leads to overdiagnosis. The number of measurements made at the time of the survey, be it on the same day or on different days, impacts significantly the prevalence of the disease. Another study that was decided to be included on the findings reported is the study of heart diseases of San Antonio (ECSA), which includes Mexican-American population and has a branch in Mexico City: the study of diabetes in Mexico City (EDCM). In these studies, the prevalence and incidence of hypertension and other cardiovascular factors are also reported.

### 1.4. Arterial Hypertension

Hypertension may be essential (unknown etiology, but with hereditary background) or secondary (with demonstrable cause) and can also be isolated or as metabolic syndrome; this disease is incapacitating and deadly due to the damage caused to important organs: blood vessels, heart, kidney, and eyes. Normal levels of blood pressure are those below 139/89 mmHg; secondary hypertension can be suspected in young people younger than 35 years of age or when there is no hypertension history in the family or in the absence of a family. Treatment of hypertension helps reducing damage to organs or even reverses it if possible; this treatment may be a drug with antidepressant use or nonpharmacological treatment, which includes changes in hygienic-dietetic habits (reduction weight, stop smoking, and drinking alcoholic beverages).

#### 1.4.1. Development of Systolic and Diastolic Hypertension

The risk of cardiovascular complications begins, apparently with blood pressure values of 115mmHg for systolic and 75 mmHg for diastolic. In the clinical area, several subtypes of hypertension determined by isolated elevations of systolic and diastolic, or the combination of both are used. These subgroups have their own natural history and present a different cardiovascular risk.

Isolated systolic hypertension (ISH) is common after 50 years of age, affecting nearly 50% of people between 50 and 59 years of age, reaching 90% in those over 80 years old. This subtype of hypertension reflects the increase in the stiffness of the aorta and great vessels without an increase in arteriolar resistance.

When there is an increase in arteriolar resistance combined with a lack of increased arterial stiffness, the isolated diastolic pressure subtype (IDP) occurs; this subtype predominates in people younger than 40, comprising almost 60% of the population [36].

#### 1.4.2. Pulse Pressure

Hypertension includes calculating pulse pressure (PP), which is done by subtracting the diastolic pressure and systolic quantities [37, 38]. In the elderly, increased systolic blood pressure reflects an increase in the degree of stiffness of arteries; as a result, pulse pressure increases. This is related to an increased incidence of cardiovascular events. Blood pressure PP is closely related to the changes produced by age in people over 50, increasing diastolic coronary mortality rates, and after 60 they stop. To most people, pulse and systolic pressure values become the most important risk triggers.

Previously, the pulse was measured when the examiner or physician would sit comfortably on the right side to support the patient's elbow and, with his right thumb, explore the antecubital fosse, where the brachial artery is. The patient's arm reflex should be activated. When the thumb or finger of the examiner is correctly in place, he or she can raise or lower the patient's forearm by varying the pressure applied to the artery, feeling the maximum pulse. The right thumb of the examiner can feel the patient's carotid artery similarly, which he or she may feel by gently grasping the patient's fingertips with theirs. The digital pulse can be counted exactly by simultaneously palpating the radial artery while the examiner supports the patient's wrist. The femoral pulse of a child of tender age should be sought only while the leg is relaxed voluntarily.

The arteries pulsations provide information about heart rhythms and speed, arterial pulse differential (right and left limbs, or top and bottom), thrills (shudders), and waveform. [39, 40].

*Bradycardia.* The bradycardia term simply means slower rate. Bradycardia athletes: the heart of an athlete is much more powerful than a normal person, which allows their heart force a greater volume of blood with each beat, a large proportion of blood driven into the arterial tree with each beat probably produces sufficient circulatory reflexes to begin causing bradycardia.

*Tachycardia.* Tachycardia means rapid heart rate. The three causes of tachycardia are increased body temperature and heart stimulation by the sympathetic and toxic states of the heart. Increased heart rate of about 10 beats per minute for each degree Celsius increases body temperature, up to 41°C; at this temperature, the heart rate may actually decrease by increasing muscle wasting as a result of fever. Tachycardia causes hyperthermia and increases the frequency of the heart rhythm [41–45].

### 1.5. Ambulatory Blood Pressure Monitoring

Nowadays modern laboratory methods often require outpatient-monitoring equipment to measure a variety of the indicators for blood pressure (BP), for 24 hours continuously. The biological rhythms are physiological functions and pathological alterations. The BP with an average heart rate of 72 beats per minute and 103.680 pulse waves is produced with corresponding changes in BP [46]. While at first the method was used in research studies, it is now increasingly used in clinical practice, as it provides additional data of measures from office and home. Moreover, only the ABMP can shed some light on symptomatic episodes occurring within 24 hours, either by raising or lowering the BP [47]. This means that it can be used not only for diagnosis of arterial hypertension (HA), but also to evaluate the frequency and severity of acute episodes of hyper- or hypotension. The ABMP is very useful to investigate the effects of new drugs for a period of 24 hours.

## 2. The Proposed Method

This section presents the proposed method for blood pressure classification, which consists of designing of modular neural networks (MNN) and the integration of responses of MNN with an average method. The main goals are to implement and find the best MNN architecture; the MNN consists of three modules; the first module is for the systolic pressure, the next module is the diastolic pressure, and in the last module we have pulse, this way we classify the arterial hypertension of a person.

**Figure 1:**
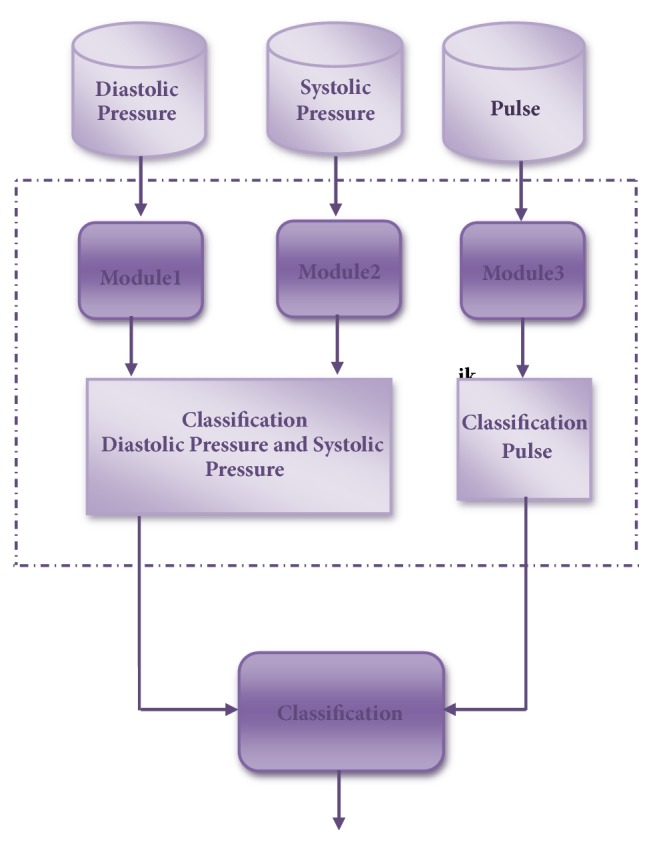
General scheme of the method.

Figure 1 illustrates the MNN structure, which considers the diastolic, systolic pressure, and the pulse for the MNN inputs, and in this case has 3 modules. The tests are performed by changing the number of layers that are between 1 and 3 and the number of neurons from 1 to 50, and in this way we obtained the responses of the MNN and integrate them with the average integration method and we obtained the classification of blood pressure.

**Figure 2:**
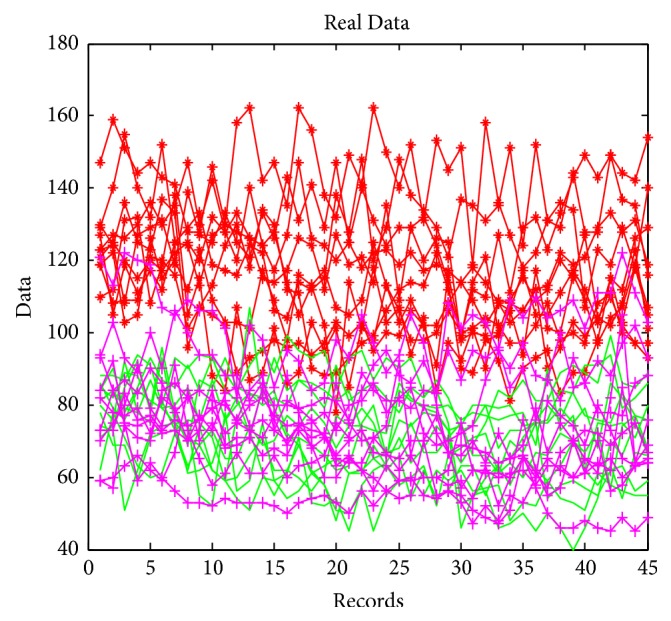
Real data of the patients.

In Figure 2, we present the data used for the classification of the arterial hypertension, where 300 patient samples are used for training all the modules in the modular neural network and we considered other 40 patients for tests with 45 records for each patient in the complete architecture.

Table 1 presents the parameters of the MNN that are manually changed to obtain the best architecture.

Table 2 presents the classification of arterial hypertension according to the European guidelines.

## 3. Discussion and Results

The proposed method to classify the blood pressure of a patient was validated with tests performed on 16 patients and positive results were obtained for the MNN.

**Figure 3:**
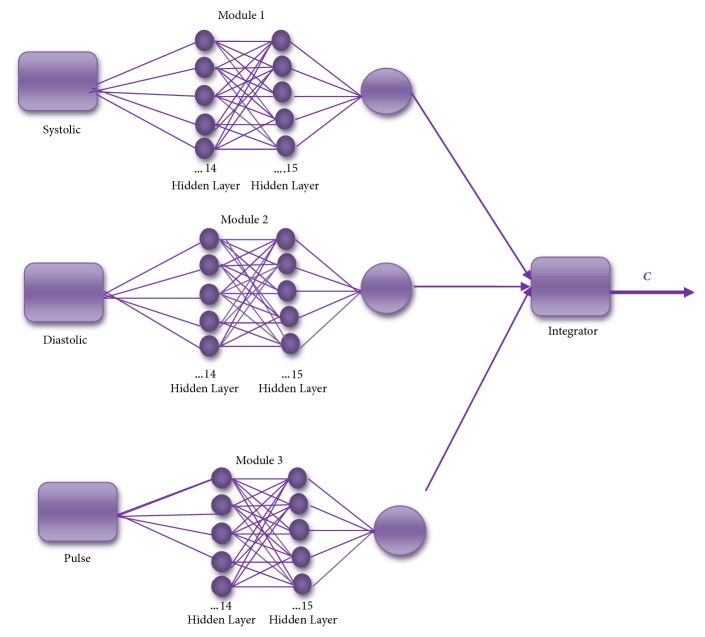
The best architecture for MNN with the training method (trainlm).

The results of the best MNN architecture are shown in Figure 3. For each of the modules of the MNN, the goal error was of 0.002 and 500 epochs were used; the number of neurons used was 14 in the first layer and 15 in the second layer.

Table 3 presents the results of the MNN with the “trainlm” method for the classification arterial hypertension.

Table 4 presents the average of the test of the MNN for each of the patients.

**Figure 4:**
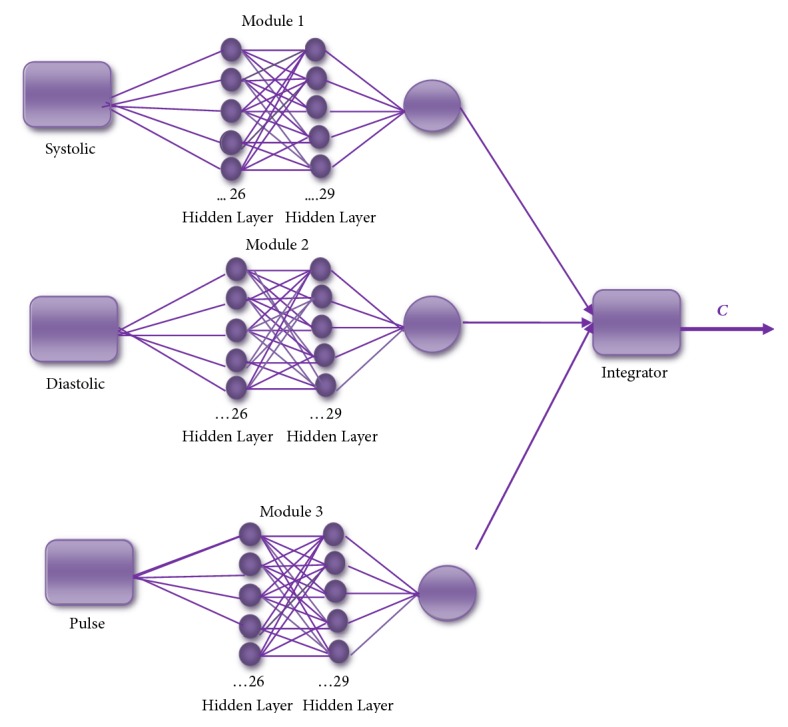
The best architecture for MNN with the training method (traincsg).

The results of the best MNN architecture are shown in Figure 4. For each of the modules of the MNN, the goal error was of 0.002 and 500 epochs were use;, the number of neurons used was 26 in the first layer and 29 in the second layer.

Table 5 presents the results of the MNN with the “trainscg” method for the classification of arterial hypertension.

In Table 6 the average of the test of the MNN for each of the persons is presented.

**Figure 5:**
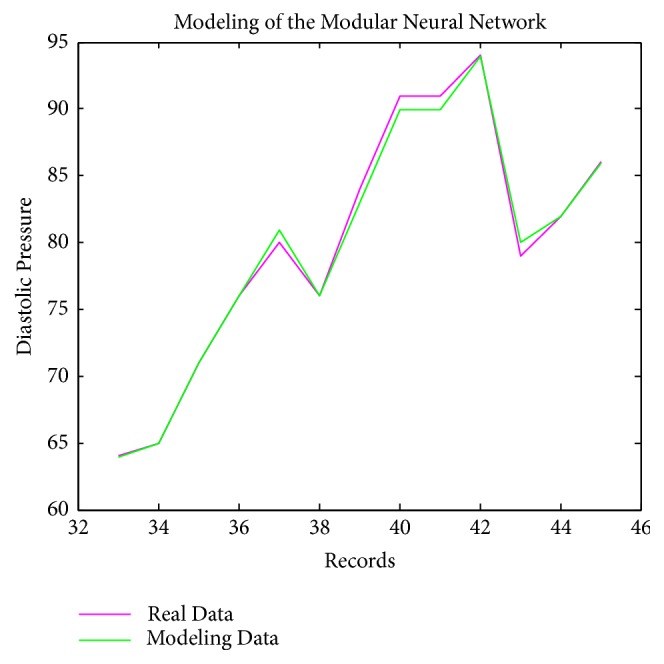
Modeling of diastolic pressure with the MNN.

Figure 5 presents the modeling data of diastolic pressure for the MNN; the pink line represents the real data and the green line represents data modeled with the MNN. The results of this model for the diastolic pressure that were obtained were good with respect to the records used to use the tests, since the trend according to the cardiologist was good.

**Figure 6:**
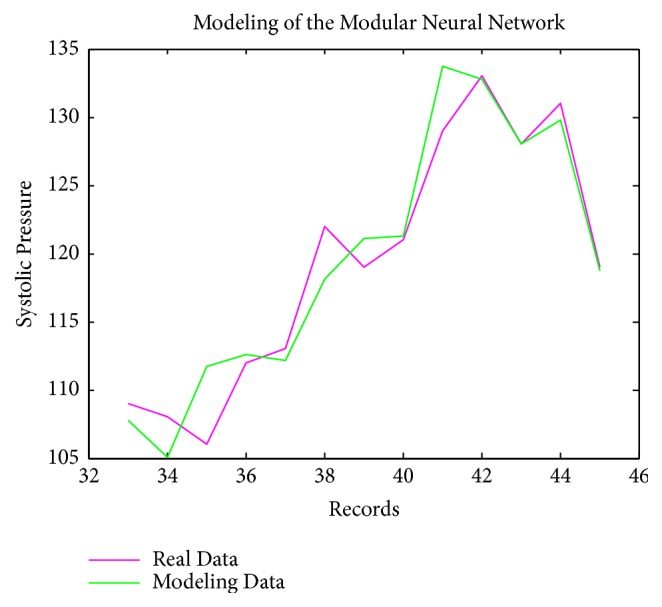
Modeling of systolic pressure with the MNN.

Figure 6 presents the modeling data of systolic pressure with the MNN proposed; the pink line represents the real data and the green line represents data modeled with the MNN. The results of this model for the systolic pressure that were obtained were good with respect to the records used to use the tests, since the trend according to the cardiologist was good.

**Figure 7:**
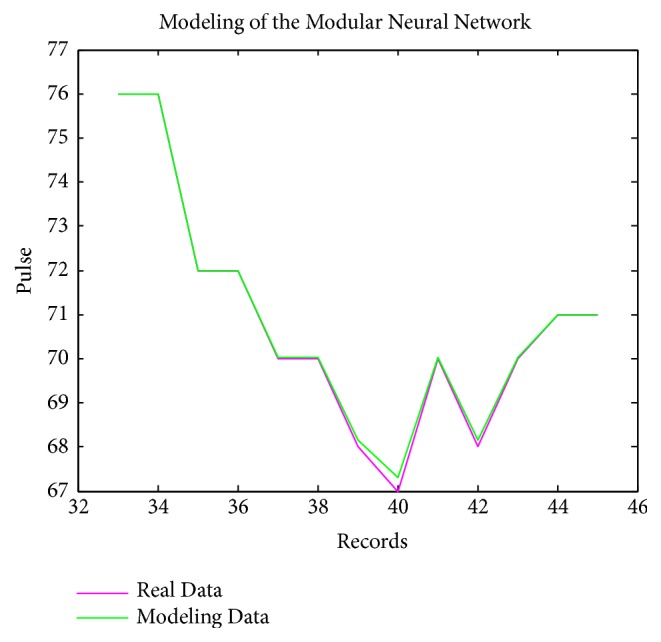
MNN modeling of pulse pressure.

Figure 7 present the modeling data of pulse pressure with the MNN; the pink line represents the real data and the green line represents data modeled with the modular neural network. The obtained results of this model for the pulse pressure were good with respect to the records used in the tests, since the trend according to the cardiologist is good.

## 4. Statistical Comparative Study

In this section a hypothesis test is made based on the errors obtained with the architecture of the modular network using the Levenberg-Marquardt learning method (trainlm) to obtain the trend of the systolic pressure. In addition, the results are compared with linear regression models based on the obtained errors.

The model used to perform the statistical comparison was the well-known linear regression. This model describes the relationship between a dependent variable and (also known as the output or answer) as a function of one or more independent variables *X* (called predictors). The general equation corresponding to a linear regression model is as follows:

(1) $$\begin{array}{c} y=\beta_{0}+\beta_{1}X+\in_{1} \end{array}$$

where

 
*β* represents a parameter that establishes the linear relationship between variables.
 
*ϵ* represents the random error terms.
 
*X* represents the real data.
 
**y** is the variable for classification.

The formulas to estimate the beta parameter values are given by

(2) β0=y~−β1x~

(3) β1=∑xy−∑xy−nx~y~∑x2−nx~2

In this case the values for *β*o and *β*_1_ are the following (for each of the modules):

*Module 1*

 
*β*o =0.101663348532528
 
*β*_1_= 1.028385618868264

*Module 2*

 
*β*o = 0.04938271
 
*β*_1_= 1.024901397932274

*Module 3*

 
*β*o =0.565544448530641
 
*β*_1_= 1.104635529315485

A set of 30 experiments are carried out to compare the results; for this, we use the parametric Z test of two samples, which is used with the following formula:

(4) $$\begin{array}{c} Z=\frac{\left({\overset{-}{x}}_{1}-{\overset{-}{x}}_{2}\right)-\left(\mu_{1}-\mu_{2}\right)}{\sigma_{{\overset{-}{x}}_{1}-{\overset{-}{x}}_{2}}} \end{array}$$

where

 
${\overset{-}{x}}_{1}-{\overset{-}{x}}_{2}$ is the observed difference.
 
(*μ*_1_ − *μ*_2_) is the expected difference.
 
$\sigma_{{\overset{-}{x}}_{1}-{\overset{-}{x}}_{2}}$ is the standard error of the difference.

The null hypothesis establishes that the mean of the errors of the systolic neural network are greater than or equal to the average of the errors obtained by the regression, being the alternative hypothesis that the mean of the errors of the systolic neural network are lower than the average of the errors obtained by the regression; the parameters of the hypothesis test are shown in Table 7.

In Table 8 we show the descriptive statistics for this test.

Table 9 shows the results obtained by applying formula (1) for Module 1.

Since the result of the p value is lower than the level of significance alpha = 0.05, we reject the null hypothesis and accept the alternative hypothesis, so we can conclude that there is sufficient evidence with a 5% level of significance to support the claim that the means of the errors of the modular neural network for the obtaining of the systolic pressure tendency are smaller than those obtained by the regression method.

In Table 10 the descriptive statistics for Module 2 (diastolic) test is shown.

Table 11 shows the results obtained by applying formula (1) for this test.

Since the result of the p value is lower than the level of significance alpha = 0.05, we reject the null hypothesis and accept the alternative hypothesis, so we can conclude that there is sufficient evidence with a 5% level of significance to support the claim that the means of the errors of the modular neural network for the obtaining of the diastolic pressure tendency are smaller than those obtained by the regression method.

We show in Table 12 the descriptive statistics for Module 3 (pulse) test.

Table 13 shows the results obtained by applying formula (1) for the third module (pulse tendency).

Since the result of the p value is less than the level of significance alpha = 0.05, we reject the null hypothesis and accept the alternative hypothesis, so we can conclude that there is sufficient evidence with a 5% level of significance to support the claim that the means of the errors of the modular neural network for obtaining the pulse tendency are smaller than those obtained by the regression method.

When comparing the model of the modular neural network with the linear regression models by means of the z-statistic tests, we can realize that when using intelligent computing techniques, in this case the modular neural networks, we have a more efficient technique to classify the systolic and diastolic pressure and the pulse and this could help the cardiologist detect and prevent diseases in the blood.

## 5. Conclusion

In this paper we have obtained good results with the proposed model. The MNN classification model for arterial hypertension was implemented with two training methods for the modular neural network, namely, the Scale Conjugate Gradient Backpropagation (trainscg) and Levenberg-Marquardt (trainlm), and we achieved good results with the second method (trainlm). Good results were also obtained in the diastolic, systolic, and pulse models, since the trend was good with respect to the records used to perform the tests. In addition, we have made a comparison between the neural network model and the regression equations, showing that the MNN model statistically outperforms the regression model. In this paper we conclude that this classification method is effective and could help the cardiologist to detect and prevent a patient's blood pressure.

## Figures and Tables

**Table 1:** MNN data.

**Number of Layers**	**Number of Neurons**	**Epochs**	**Learning Rate**	**Error Goal**	**Training Methods**
**1 to 3**	1 to 50	500	0.001	0.01	(i) Levenberg-Marquardt (trainlm) (ii) Scaled Conjugate Gradient Back Propagation (traincsg).

**Table 2:** Blood pressure levels (mmHg).

Category	Systolic		Diastolic
Optimal	<120	And	<80

Normal	120-129	And/or	80-84

High Normal	130-139	And/or	85-89

Grade 1 Hypertension	140-159	And/or	90-99

Grade 2 Hypertension	160-179	And/or	100-109

Grade 3 Hypertension	≥180	And/or	≥110

Isolated Systolic hypertension	≥140	And/or	<90

**Table 3:** Classification for MNN with train method (trainlm).

**No. Persons**	**Number of Neurons**	**Time**	**Systolic**	**Diastolic**	**Pulse**	**Classification**
**Person1**	14,15	00:06:48	118	70	68	Optimal

**Person 2**	14,15	00:06:48	109	77	76	Optimal

**Person 3**	14,15	00:06:48	112	76	79	Optimal

**Person 4**	14,15	00:06:48	120	73	73	Optimal

**Person 5**	14,15	00:06:48	146	86	77	Grade 1 Hypertension

**Person 6**	14,15	00:06:48	107	63	92	Optimal

**Person 7**	14,15	00:06:48	128	83	97	Normal

**Person 8**	14,15	00:06:48	112	66	96	Optimal

**Person 9**	14,15	00:06:48	130	76	73	Normal

**Person 10**	14,15	00:06:48	123	79	57	Normal

**Person 11**	14,15	00:06:48	138	65	65	High Normal

**Person 12**	14,15	00:06:48	138	84	74	High Normal

**Person 13**	14,15	00:06:48	123	76	79	Normal

**Person 14**	14,15	00:06:48	114	63	78	Optimal

**Person 15**	14,15	00:06:48	124	79	72	Normal

**Person 16**	14,15	00:06:48	134	84	89	High Normal

**Person 17**	14,15	00:06:48	125	77	80	Normal

**Person 18**	14,15	00:06:48	106	65	79	Optimal

**Person 19**	14,15	00:06:48	110	68	79	Optimal

**Person 20**	14,15	00:06:48	123	76	80	Normal

**Person 21**	14,15	00:06:48	115	72	76	Optimal

**Person 22**	14,15	00:06:48	112	73	78	Optimal

**Person 23**	14,15	00:06:48	122	76	77	Normal

**Person 24**	14,15	00:06:48	117	68	90	Optimal

**Person 25**	14,15	00:06:48	121	74	92	Optimal

**Person 26**	14,15	00:06:48	130	82	89	Normal

**Person 27**	14,15	00:06:48	121	63	86	Optimal

**Person 28**	14,15	00:06:48	112	73	90	Optimal

**Person 29**	14,15	00:06:48	123	82	80	Normal

**Person 30**	14,15	00:06:48	95	61	73	Optimal

**Person 31**	14,15	00:06:48	108	65	72	Optimal

**Person 32**	14,15	00:06:48	110	70	73	Optimal

**Person 33**	14,15	00:06:48	116	67	71	Normal

**Person 34**	14,15	00:06:48	130	86	80	Normal

**Person 35**	14,15	00:06:48	117	73	80	Optimal

**Person 36**	14,15	00:06:48	117	54	81	Optimal

**Person 37**	14,15	00:06:48	113	74	74	Optimal

**Person 38**	14,15	00:06:48	132	86	79	Normal

**Person 39**	14,15	00:06:48	128	80	78	Normal

**Person 40**	14,15	00:06:48	131	85	70	Normal

**Table 4:** Average results of the MNN with (trainlm).

**Person**	**Time**	**Systolic**	**Diastolic**	**Pulse**
Person 1	00:15:34	115	73	67

Person 2	00:15:34	105	72	77

Person 3	00:15:34	114	70	80

Person 4	00:15:34	119	71	72

Person 5	00:15:34	145	84	75

Person 6	00:15:34	104	61	90

Person 7	00:15:34	125	87	96

Person 8	00:15:34	109	64	73

Person 9	00:15:34	129	73	56

Person 10	00:15:34	122	77	65

Person 11	00:15:34	136	63	70

Person 12	00:15:34	136	81	71

Person 13	00:15:34	120	74	78

Person 14	00:15:34	110	62	77

Person 15	00:15:34	119	68	70

Person 16	00:15:34	131	82	80

Person 17	00:15:34	125	77	80

Person 18	00:15:34	106	65	79

Person 19	00:15:34	110	68	79

Person 20	00:15:34	123	76	80

Person 21	00:15:34	115	72	76

Person 22	00:15:34	112	73	78

Person 23	00:15:34	122	76	77

Person 24	00:15:34	117	68	90

Person 25	00:15:34	121	74	92

Person 26	00:15:34	130	82	89

Person 27	00:15:34	121	63	86

Person 28	00:15:34	112	73	90

Person 29	00:15:34	123	82	80

Person 30	00:15:34	95	61	73

Person 31	00:15:34	108	65	72

Person 32	00:15:34	110	70	73

Person 33	00:15:34	116	67	71

Person 34	00:15:34	130	86	80

Person 35	00:15:34	117	73	80

Person 36	00:15:34	117	54	81

Person 37	00:15:34	113	74	74

Person 38	00:15:34	132	86	79

Person 39	00:15:34	128	80	78

Person 40	00:15:34	131	85	70

**Table 5:** Classification for MNN with train method (traincsg).

**No. Persons**	**Number of Neurons**	**Time**	**Systolic**	**Diastolic**	**Pulse**	**Classification**
**Person1**	26,29	00:07:12	116	72	67	Optimal

**Person 2**	26,29	00:07:12	106	72	77	Optimal

**Person 3**	26,29	00:07:12	114	70	80	Optimal

**Person 4**	26,29	00:07:12	119	71	72	Optimal

**Person 5**	26,29	00:07:12	145	84	75	Grade1 Hypertension

**Person 6**	26,29	00:07:12	107	61	90	Normal

**Person 7**	26,29	00:07:12	130	87	97	High Normal

**Person 8**	26,29	00:07:12	120	64	97	Optimal

**Person 9**	26,29	00:07:12	131	74	73	High Normal

**Person 10**	26,29	00:07:12	122	78	57	Normal

**Person 11**	26,29	00:07:12	136	63	65	High Normal

**Person 12**	26,29	00:07:12	135	75	70	High Normal

**Person 13**	26,29	00:07:12	120	75	78	Normal

**Person14**	26,29	00:07:12	117	72	77	Optimal

**Person 15**	26,29	00:07:12	121	69	70	Normal

**Person 16**	26,29	00:07:12	132	90	78	High Normal

**Person 17**	26,29	00:07:12	126	76	80	Normal

**Person 18**	26,29	00:07:12	106	65	82	Optimal

**Person 19**	26,29	00:07:12	110	68	84	Optimal

**Person 20**	26,29	00:07:12	123	76	90	Normal

**Person 21**	26,29	00:07:12	112	71	78	Optimal

**Person 22**	26,29	00:07:12	111	70	70	Optimal

**Person 23**	26,29	00:07:12	122	73	71	Normal

**Person 24**	26,29	00:07:12	116	67	80	Optimal

**Person 25**	26,29	00:07:12	120	74	80	Optimal

**Person 26**	26,29	00:07:12	129	80	79	Normal

**Person 27**	26,29	00:07:12	120	61	74	Optimal

**Person 28**	26,29	00:07:12	112	73	81	Optimal

**Person 29**	26,29	00:07:12	121	82	67	Normal

**Person 30**	26,29	00:07:12	95	61	77	Optimal

**Person 31**	26,29	00:07:12	106	65	80	Optimal

**Person 32**	26,29	00:07:12	116	75	72	Optimal

**Person 33**	26,29	00:07:12	116	71	75	Normal

**Person 34**	26,29	00:07:12	130	86	90	Normal

**Person 35**	26,29	00:07:12	117	74	97	Optimal

**Person 36**	26,29	00:07:12	117	58	88	Optimal

**Person 37**	26,29	00:07:12	113	70	73	Optimal

**Person 38**	26,29	00:07:12	131	71	80	Normal

**Person 39**	26,29	00:07:12	128	81	82	Normal

**Person 40**	26,29	00:07:12	134	85	81	Normal

**Table 6:** Average of the MNN with (traincsg).

Person	Time	Systolic	Diastolic	Pulse
Person 1	00:17:19	115	73	67

Person 2	00:17:19	105	72	77

Person 3	00:17:19	114	70	80

Person 4	00:17:19	119	71	72

Person 5	00:17:19	145	84	75

Person 6	00:17:19	104	61	90

Person 7	00:17:19	125	87	96

Person 8	00:17:19	109	64	73

Person 9	00:17:19	129	73	56

Person 10	00:17:19	122	77	65

Person 11	00:17:19	136	63	70

Person 12	00:17:19	136	81	71

Person 13	00:17:19	120	74	78

Person 14	00:17:19	110	62	77

Person 15	00:17:19	119	68	70

Person 16	00:17:19	131	82	80

Person 17	00:17:19	126	76	80

Person 18	00:17:19	106	65	82

Person 19	00:17:19	110	68	84

Person 20	00:17:19	123	76	90

Person 21	00:17:19	112	71	78

Person 22	00:17:19	111	70	70

Person 23	00:17:19	122	73	71

Person 24	00:17:19	116	67	80

Person 25	00:17:19	120	74	80

Person 26	00:17:19	129	80	79

Person 27	00:17:19	120	61	74

Person 28	00:17:19	112	73	81

Person 29	00:17:19	121	82	67

Person 30	00:17:19	95	61	77

Person 31	00:17:19	106	65	80

Person 32	00:17:19	116	75	72

Person 33	00:17:19	116	71	75

Person 34	00:17:19	130	86	90

Person 35	00:17:19	117	74	97

Person 36	00:17:19	117	58	88

Person 37	00:17:19	113	70	73

Person 38	00:17:19	131	71	80

Person 39	00:17:19	128	81	82

Person 40	00:17:19	134	85	81

**Table 7:** Parameters for hypothesis testing modules.

Parameters	
Confidence Interval	95%

Alfa	0.05

Ho	*μ*_1_ ≥ *μ*_2_

Ha	*μ*_1_ < *μ*_2_

Critical Value	Z= -1.645

**Table 8:** Descriptive statistics for Module 1 (systolic).

Variable	Observations	Mean	Std. Derivation
MNN(sys)	30	9.820	1.997

Regression	30	16.830	4.508

**Table 9:** Results of the Z-test for Module 1.

Difference	-7.010
z (Observed Value)	-7.788

z (Critical Value)	-1.645

p-value	<3.33066907387547x10^−15^

Alfa	0.05

**Table 10:** Descriptive statistics Module 2 (diastolic).

Variable	Observations	Mean	Std. Derivation
MNN(sis)	30	23.177	3.096

Regression	30	34.778	5.438

**Table 11:** Results of the Z-test for Module 2.

Difference	-9.462
z (Observed Value)	-8.2383

z (Critical Value)	-1.645

p-value	<1.11022302462516x10^−16^

Alfa	0.05

**Table 12:** Descriptive statistics Module 3 (pulse).

Variable	Observations	Mean	Std. Derivation
MNN(sis)	30	14.774	1.821

Regression	30	28.367	4.733

**Table 13:** Results of the Z-test Module 3.

Difference	-13.593
z (Observed Value)	-14.682

z (Critical Value)	-1.645

p-value	<2.59524148975464x10^−17^

Alfa	0.05

## Data Availability

The data that was used in this research to support the findings of this study are available from the corresponding author upon request by email pmelin@tectijuana.mx.

## References

[1] Abdullah A. A., Zakaria Z., Mohammad N. F. Design and development of fuzzy expert system for diagnosis of hypertension.

[2] Abdullah A. A., Zakaria Z., Mohammad N. F. Design and development of fuzzy expert system for diagnosis of hypertension.

[3] Kaur R., Kaur A. (2014). Hypertension Diagnosis Using Fuzzy Expert System. *International Journal of Engineering Research and Applications (IJERA)*.

[4] Poli R., Cagnoni S., Coppini G., Valli G. (1991). A Neural Network Expert System for Diagnosing and Treating Hypertension. *The Computer Journal*.

[5] Fuller R., Giove S. A Neuro-Fuzzy Approach to FMOLP Problems.

[6] Nohria R., Mann P. S. (2015). Diagnosis of Hypertension using Adaptive Neuro-Fuzzy Inference System. *IJCST*.

[7] Zeinab A., Hamid T. (2015). *Design of a Fuzzy Expert System and A Multi-layer Neural Network System for Diagnosis of Hypertension*.

[8] Yafawi R., Knauft M. E., Stokem K., Palminteri J. M., WirthJ M. (2018). Pulmonary arterial hypertension. *Encyclopedia of Cardiovascular Research and Medecine*.

[9] Kallistratos M. S., Poulimenos L. E., Manolis A. J. (2018). Atrial fibrillation and arterial hypertension. *Pharmacological Research*.

[10] Cuspidi C., Tadic M., Grassi G., Mancia G. (2018). Treatment of hypertension: The ESH/ESC guidelines recommendations. *Pharmacological Research*.

[11] Corrado A., Correale M., Mansueto N. (2017). Nailfold capillaroscopic changes in patients with idiopathic pulmonary arterial hypertension and systemic sclerosis-related pulmonary arterial hypertension. *Microvascular Research*.

[12] Srivastava P., Srivastava A., Burande A., Khandelwal A. (2013). A note on hypertension classification scheme and soft computing decision making system. *ISRN Biomathematics*.

[13] Huang S., Xu Y., Yue L. (2010). Evaluating the risk of hypertension using an artificial neural network method in rural residents over the age of 35 years in a Chinese area. *Hypertension Research*.

[14] Srivastava P., Srivastava A., Burande A., Khandelwal A. (2013). A note on hypertension classification scheme and soft computing decision making system. *ISRN Biomathematics*.

[15] Ture M., Kurt I., Turhan Kurum A., Ozdamar K. (2005). Comparing classification techniques for predicting essential hypertension. *Expert Systems with Applications*.

[16] Melin P., Miramontes I., Prado-Arechiga G. (2018). A hybrid model based on modular neural networks and fuzzy systems for classification of blood pressure and hypertension risk diagnosis. *Expert Systems with Applications*.

[17] Melin P., Prado-Arechiga G. (2018). *New Hybrid Intelligent Systems for Diagnosis and Risk Evaluation of Arterial Hypertension*.

[18] Guzman J. C., Melin P., Prado-Arechiga G. (2017). Design of an optimized fuzzy classifier for the diagnosis of blood pressure with a new computational method for expert rule optimization. *Algorithms*.

[19] Das S., Ghosh P. K., Kar S. Hypertension diagnosis: A comparative study using fuzzy expert system and neuro fuzzy system.

[20] Djam X. Y., Kimbi Y. H. (2011). Fuzzy expert system for the management of hypertension. *Pacific Journal of Science and Technology*.

[21] Klabunde R. E. (2011). *Cardiovascular Physiologic Concepts*.

[22] Ludmila I. K., Steimann F. (2008). *Fuzzy Medical Diagnosis, School of Mathematics*.

[23] Zhang H., Lin F. C. (1999). Medical Diagnosis by the Virtual Physician. *IEEE Xplore Computer Based Medical System*.

[24] Clec'h Y., Vicaut C., Marbeuf-G E. (2009). Can fuzzy logic make things more clear?. *Critical Care*.

[25] Grübler M. R., Gaksch M., Kienreich K. (2018). Effects of Vitamin D3 on asymmetric- and symmetric dimethylarginine in arterial hypertension. *The Journal of Steroid Biochemistry and Molecular Biology*.

[26] Sumathi B., Santhakumaran A. (2011). Pre-diagnosis of hypertension using artificial neural network. *Global Journal of Computer Science and Technology, Coimbator, Tamil Nadu*.

[27] Vilkov V. G., Oganov R. G., Shal'nova S. A. (2006). Comparative accuracy of neural network models for diagnosing latent arterial hypertension on the basis of data on daily blood pressure monitoring. *Human Physiology*.

[28] Kaur A., Bhardwaj A. (2014). Genetic neuro fuzzy system for hypertension diagnosis. *International Journal of Computer Science and Information Technologies*.

[29] Barman M., Choudhury J. (2012). A fuzzy rule base system for the diagnosis of heart disease. *International Journal of Computer Applications*.

[30] Patil P., Mohsin S. (2013). Fuzzy Logic based Health Care System using Wireless Body Area Network. *International Journal of Computer Applications*.

[31] Morsi I., Abd El Gawad Y. Z. Fuzzy logic in heart rate and blood pressure measuring system.

[32] Hosseini S., Jutten C., Charbonnier S. (2003). Neural network modeling of ambulatory systolic blood pressure for hypertension diagnosis. *Artificial Neural Nets Problem Solving Methods*.

[33] Touyz R. M., Lang N. N., Herrmann J., Van Den Meiracker A. H., Danser A. H. J. (2017). Recent Advances in Hypertension and Cardiovascular Toxicities With Vascular Endothel ial Growth Factor Inhibition. *Recent Advances in Hypertension*.

[34] Feng L., Khan A. H., Jehan I., Allen J., Jafar T. H. (2017). Albuminuria and kidney function as prognostic marker of left ventricular mass among South Asians with hypertension. *Journal of the American Society of Hypertension*.

[35] Nierenberg J. L., Li C., He J. (2017). Blood Pressure Genetic Risk Score Predicts Blood Pressure Responses to Dietary Sodium and Potassium: The GenSalt Study (Genetic Epidemiology Network of Salt Sensitivity). *Hypertension (Dallas, Tex. : 1979)*.

[36] Jang J. S. R., Sun C. T., Mizutani E. (1996). *Neuro-Fuzzy and Soft Computing*.

[37] Torlasco C., Faini A., Makil E. (2017). Cardiovascular risk and hypertension control in Italy. Data from the 2015 World Hypertension Day. *International Journal of Cardiology*.

[38] Beevers G., Lip G. Y. H., O'brien E. (2001). Blood pressure measurement: Part I—Sphygmomanometry: Factors common to all techniques. *BMJ*.

[39] Bernstein D., Kliegman R. M., Stanton B. F., St. Geme J. W. (2015). Evaluation of the cardiovascular system: history and physical. *Evaluation*.

[40] Harrison (2006). Tachyarrhythmias. *Principles of Internal Medicine*.

[41] American Heart Association. https://www.heart.org/.

[42] Simel D. L., Goldman L., Schafer A. I. (2012). Approach to the Patient: History and Physical Examination. *Goldman's Cecil Medicine*.

[43] (2003). World Health Organization. *International Society of Hypertension Group*.

[44] Whitworth J. A. (2003). World Health Organization (WHO)/International Society of Hypertension (ISH) statement on management of hypertension. *Journal of Hypertension*.

[45] Parati G., Mancia G., Mancia G., Chalmers J., Julius S. (2002). Ambulatory Blood Pressure Monitoring. *Manual of Hypertension*.

[46] Sokolow M. (1993). Ambulatory blood pressure a personal historical account. *American Journal of Hypertension*.

[47] Parati G., Mancia G. (2002). Ambulatory blood pressure monitoring in clinical practice. *Journal of Hypertension*.

